# Climate-Fungal Pathogen Modeling Predicts Loss of Up to One-Third of Tea Growing Areas

**DOI:** 10.3389/fcimb.2021.610567

**Published:** 2021-04-29

**Authors:** Saowaluck Tibpromma, Yang Dong, Sailesh Ranjitkar, Douglas A. Schaefer, Samantha C. Karunarathna, Kevin D. Hyde, Ruvishika S. Jayawardena, Ishara S. Manawasinghe, Daniel P. Bebber, Itthayakorn Promputtha, Jianchu Xu, Peter E. Mortimer, Jun Sheng

**Affiliations:** ^1^ CAS Key Laboratory for Plant Diversity and Biogeography of East Asia, Kunming Institute of Botany, Chinese Academy of Science, Kunming, China; ^2^ World Agroforestry Centre, East and Central Asia, Kunming, China; ^3^ Centre for Mountain Futures, Kunming Institute of Botany, Kunming, China; ^4^ State Key Laboratory for Conservation and Utilization of Bio-Resources in Yunnan, Yunnan Agricultural University, Kunming, China; ^5^ Yunnan Research Institute for Local Plateau Agriculture and Industry, Kunming, China; ^6^ N. Gene Solution of Natural Innovation, Kathmandu, Nepal; ^7^ Center of Excellence in Fungal Research, Mae Fah Luang University, Chiang Rai, Thailand; ^8^ Department of Biosciences, University of Exeter, Exeter, United Kingdom; ^9^ Department of Biology, Faculty of Science, Chiang Mai University, Chiang Mai, Thailand; ^10^ Research Center in Bioresources for Agriculture, Industry and Medicine, Chiang Mai University, Chiang Mai, Thailand; ^11^ Key Laboratory for Agro-biodiversity and Pest Control of Ministry of Education, Yunnan Agricultural University, Kunming, China

**Keywords:** *Camellia sinensis*, climate change, crop loss, fungal diseases, perennial crops

## Abstract

Climate change will affect numerous crops in the future; however, perennial crops, such as tea, are particularly vulnerable. Climate change will also strongly influence fungal pathogens. Here, we predict how future climatic conditions will impact tea and its associated pathogens. We collected data on the three most important fungal pathogens of tea (*Colletotrichum acutatum*, *Co. camelliae*, and *Exobasidium vexans*) and then modeled distributions of tea and these fungal pathogens using current and projected climates. The models show that baseline tea-growing areas will become unsuitable for *Camellia sinensis* var. *sinensis* (15 to 32% loss) and *C. sinensis* var. *assamica* (32 to 34% loss) by 2050. Although new areas will become more suitable for tea cultivation, existing and potentially new fungal pathogens will present challenges in these areas, and they are already under other land-use regimes. In addition, future climatic scenarios suitable range of fungal species and tea suitable cultivation (respectively in CSS and CSA) growing areas are *Co. acutatum* (44.30%; 31.05%), *Co. camelliae* (13.10%; 10.70%), and *E. vexans* (10.20%; 11.90%). Protecting global tea cultivation requires innovative approaches that consider fungal genomics as part and parcel of plant pathology.

## Introduction

Climatic changes can alter fungal survivability and infectivity as well as host susceptibility, leading to new disease outbreaks (Elad and Pertot, 2014; Cheng et al., 2019). They may also facilitate the emergence of new virulent strains, a cause of significant concern for future epidemiological research (Garrett et al., 2006; Chen et al., 2019). Several studies on fungal diseases and climate change have revealed that the number of fungal diseases has increased alongside climate change, and it is increasingly recognized as a worldwide threat to important crops (Fisher et al., 2012). Bebber et al. (2013) found that fungal pathogens are moving poleward at 7.61 ± 2.41 km/y in the Northern Hemisphere, while climates move at about 2.7 km/y. Trade and transport are likely to be accelerating the spread of fungal pathogens. With land-use change, drought, and climate variability, fungi need adaptation or shifts in community for survival, while some fungi with higher thermal optima have higher fitness for climate change (McLean et al., 2005; Bajpai and Johri, 2019). Consequently, tea as perennial plant might not escape fungal threats unless it is relocated.

Several studies have been conducted to gain insight into the responses of perennial crops such as almonds, avocados, grapes, and walnuts to climate projections (Lobell et al., 2006). Diseases in perennial crops have been well researched, but the impact of climate change on the distribution of these fungal diseases on tea has not yet been studied (Karakaya and Dikilitas, 2018; Nowogrodzki, 2019). Tea comprises almost one-quarter of the global perennial crop market, including coffee, tree fruits, and tree nuts (**Supplementary Table S1**). There are two main varieties of cultivated tea: *Camellia sinensis* var. *sinensis* (CSS) and *Camellia sinensis* var. *assamica* (CSA) (Ahmed and Stepp, 2013). The difference of the two tea varieties can be distinguished: var. *assamica*, quick-growing with a large leaf, tall and well suited to very warm tropical climates, and with high sensitivity to cold weather, while var. *sinensis*, lower-growing shrubs, with small leaves, and able to withstand colder climates (Kaundun and Matsumoto, 2003; Ming and Bartholomew, 2007). Globally, tea is usually grown in monocultural plantations in humid climates, where genetic uniformity and low environmental variability facilitate the spread of fungal pathogens. In Yunnan (China), tea is grown in between forest, agroforest, and mixed-crop (Lehmann-Danzinger, 2000; Ahmed et al., 2010; Diez et al., 2013).

Fungal pathogens that affect tea plant leaves can lead to a significant reduction in their quantity and quality, resulting in a loss of revenue (Gulati et al., 1993; Baby, 2002; Wang L. et al., 2016; Wang Y. et al., 2016; Cheng et al., 2019; Sun et al., 2019). Approximately 507 fungal pathogenic species are associated with tea plants (Chen and Chen, 1990), and among them *Exobasidium vexans* mainly attacks tea leaves which make ~40% yield loss (Sun et al., 2019; Chaliha and Kalita, 2020). Some studies investigated the effects of future climates on the distribution of other perennial crops, including coffee in Ethiopia (Moat et al., 2017), grapevines in the United Kingdom (Mosedale et al., 2015), and cultivated grasses in Sichuan, China (Guo et al., 2014). Juroszek et al. (2020) reviewed the potential climate change effects on plant pathogens and crop disease risks during the past 30 years, but tea was not included. Modeling land with highly, moderately, and non-suitable regions for the cultivation of tea crops in Zhejiang, China under baseline climates was done by Li et al. (2012). However, this study did not include global land suitability and the impacts of fungal diseases on tea crops. Several regional studies examined climate change effects on tea yield and/or quality and also used regression models to predict tea crop yields (Wijeratne et al., 2007; Patra et al., 2013; Karunaratne et al., 2015; Boehm et al., 2016; Duncan et al., 2016; Gunathilaka et al., 2016; CIAT, 2017; Sitienei et al., 2017; Ahmed et al., 2018; Gunathilaka et al., 2018; Han et al., 2018; Zakir, 2018; Ahmed et al., 2019). Karakaya and Dikilitas (2018) dealt particularly with the biochemical, physiological, and molecular defense mechanisms of tea plants against pathogenic fungi under changing climate conditions.

None of these studies considered climate change in the context of the effects of fungal pathogens on perennial crops like tea. Ecological niche modeling (ENM) based prediction of suitable habitat for host and pathogen could provide baseline infromation on how pathogens would spread in potential tea plantation regions in the future. Therefore, we aim to generate distribution models of a host crop and its fungal pathogens to enhance understanding of how these pathogens might spread and impact potential tea plantation regions under future climatic scenarios. Also gaps in this research include changes across global plantations in the two most common commercial varieties of tea.

We reviewed the literature on where cultivated tea is globally affected by fungal pathogens and global climate projections to anticipate future tea climates as well as future tea-fungal pathogen climates (Fick and Hijmans, 2017; Větrovský et al., 2019). Baseline (baseline scenario) and future climate scenarios (2050) were used for generating suitability mapping for suitability for pathogens under current conditions with regional and global climate projections. This tea-pathogen scenario is only a case study which can be applied to many different host–pathogen distributions in different disciplines. In addition, we give pattern of spore and appressorium germination of *Co. acutatum* at growth rate peaks 25 to 27.5°C.

## Methods

We obtained data from published literature, online databases, and personnal communication. Most fungal pathogenic reports in tea originated from China and India. Bioclimatic correlations were the main factors that we used to model probability of occurrence among these pathogens. Distributions of fungal pathogens were linked to bioclimatic factors, particularly their unique temperature and precipitation requirements. We collected fungal pathogen data from “Google Scholar”, “Research Gate”, and “Web of Science”. The main keywords used in the online literature search information regarding distribution of tea and tea pathogens were: “*Camellia sinensis*”, “fungal pathogen”, and “tea plantation”. Those following discovery of tea pathogens were most importantly fungal tea disease and distribution of tea. Based on the review of relevant literature, a list of tea fungal pathogens was prepared. Distribution of tea occurrence records was compiled in several locations, and final data were used for the analyses of current and projected climates. Tea leaf sample data (280 from Africa and 654 samples from Asian countries) were obtained from Global Biodiversity Information Facility (GBIF), while tea samples from America and Australia were obtained from online website (www.killgreen.io/main/us-grown-tea; www.youtube.com/c/CuppaChaTEA). Sampling includes collections from tea plantations as well as wild populations especially in China. Tea growers in Asian countries were contacted afterwards to find out from where tea leaves were collected and to get information of pathogens in tea plantations. Therefore, for comparative purposes in the case of diseases on tea, the global geographic distribution of fungal tea pathogens was summarized from existing literature and combined with information on major fungal diseases as well as the evolution of this research field. In addition, species identity of the selected fungal diseases was confirmed by published literature that was based on morphology, genomics, and pathogenicity (**Supplementary Table S2**). Three major fungal diseases on tea were selected, and these caused diseases on more than one host species. In addition, we obtained tea and the fungal pathogen distribution records from GBIF. This database includes information taken from the CABI database. In addition, we also have ground truthing data for tea that were recorded in 2013/14. The leaf sample collection records used as ground-based data come from Bangladesh, Cameroon, China, India, Kenya, Madagascar, Malawi, Nepal, Nigeria, Pakistan, Rwanda, South Africa, Sri Lanka, and Tanzania.

Based on information available on disease incidence at various geographical locations, we selected three important fungal pathogens on tea leaves that are briefly described here. The genus *Colletotrichum* represents high diversity, and several species are plant pathogens that cause diseases in many economically important crops. In this study, we selected two species of *Colletotrichum* (*Co. acutatum* and *Co. camelliae*) that are either already known to cause disease or are considered as emergent diseases on the tea plant. *Colletotrichum acutatum* is a new causal agent for brown blight on tea, which is generally considered as an endophytic fungus but is also considered pathogenic or potentially pathogenic on tea. *Colletotrichum camelliae* is the earliest known cause of tea anthracnose, and it is notable that this pathogen has been reported only from tea, and knowledge of it is limited. *Exobasidium vexans* is an obligate pathogen capable of attacking young leaves and is recognized as the most serious disease in cultivated tea. Considering the data on tea leaf-affecting pathogens, we selected *Co. acutatum*, *Co. camelliae*, and *E. vexans* for our modeling. The three pathogens used in our modeling process were selected by occurring wide range of crops in tropical and subtropical areas, also latent infection on tea (*Co. acutatum*, *Co. camelliae*) with mainly pathogen only in tea (*E. vexans*). We includeed *Co. acutatum* because this species was treated as a regulated quarantine pest by the European and Mediterranean Plant Protection Organization (EPPO) for many years until 2011 (EPPO, 2011). This species in a broad sense is a suitable candidate pathogen for modeling disease development on tea in the context of climate change because there are economically important pathogens in tea that infect tea leaves which is the most economically important part of tea (Peres et al., 2005; Wharton and Schilder, 2008; Chen et al., 2016; Chen et al., 2017). In addition, several fungal pathogens on tea are shown in **Supplementary Table S2**. These three taxa are obligate parasites, highly pathogenic, and responsible for crop losses (Dean et al., 2012; Wang Y. et al., 2016; Wang L. et al., 2016; Li et al., 2017).

### Bioclimatic Data

We obtained 19 biologically significant climate variables (bioclim) from Wordclim version 2 (www.worldclim.org/version2) (Fick and Hijmans, 2017) to generate distribution models. For future projections of both tea and fungal models, bioclimatic variables (monthly temperature and precipitation), tea and fungal models were derived from the results of 19 Earth System Models (ESM) provided by the Coupled Model Intercomparison Project-Phase 5 (CMIP5) (Taylor et al., 2012). Relative humidity data were used for tea and pathogenic distribution, as relative humidity and temperature were main factors affecting pathogens (Jones and Wint, 2015). There are four representative concentration pathways (RCP) (Van Vuuren et al., 2011) within each of the 19 ESM, ranging from RCP 2.6 (aggressive mitigation/lowest emissions) to RCP 8.5 (highest emissions scenario). All models available within each RCP were combined into a majority ensemble result (E26, E45, E60 and E85) (Zomer et al., 2015; Ranjitkar et al., 2016a). Grid cell of all the climatic layers used was adjusted to 0.0833 (~10 km spatial resolution). Tea location data was prepared with approximately 20 km spatial resolution of presence data where each 20 km^2^ grid has a single point of present data. The rest of the data points, if available within 20 km^2^ grid were discarded to minimize spatial autocorrelation and possible overfitting of the model output maps (Ranjitkar et al., 2014). Tea and pathogen data were extracted from tea plantation areas and pathogen incidence recordings. Information obtained from temporal distribution and climatic range was used to select appropriate bioclimate thresholds (temperature and precipitation) to project bioclimatic suitability for the pathogens. Out of these, temperature is the most vital factor affecting fungal growth that could cease at non-permissive temperatures.

In addition, data of optimum temperatures for conidia germination, appressoria formation, and anthracnose development of pathogens (*Co. acutatum* and *Co. gloeosporioides*) were obtained from available literature (Kenny et al., 2012). Temperature induced fungal pathogen growth with percent germination, appressorium, and anthracnose disease was used to genetate graph with fungal growth, in that range (**Supplementary Figure S1**).

### Habitat Suitability Modeling

MaxEnt 3.4.1 (Phillips et al., 2004; Phillips et al., 2006; Phillips and Dudík, 2008) was used for generating suitability maps for tea and pathogens in the baseline (baseline scenario) and future (2050) climate scenarios. Presence data and pseudoabsence were used, and prediction model was created using MaxEnt software. To make the model robust to current tea cultivation area, we selected pseudoabsence points using a 100 km radius of available presence data (Senay et al., 2013; Ranjitkar et al., 2014; Warren et al., 2020). Altogether, 20,000 pseudoabsence points were selected. Bioclimatic factors for both species occurrence and pseudoabsence data were extracted and used for running the model in Maxent. Altogether 20 replicates of the model with 19 bioclimatic variables (obtained from the worldclim database) were run setting MaxEnt to select 75% of occurrence localities randomly for calibration and the remaining 25% for evaluation after each run. The bootstrap method was used with maximum iteration = 5,000, convergence threshold = 0.0001, maximum background points = 20,000 and default features. Initial modeling results were thoroughly checked and high contributions and important variables were selected and retained while other highly correlated variables were removed using variance inflation factor (VIF) statistics. Highly correlated variables were indicated by VIF <10 (Ranjitkar et al., 2016a; Ranjitkar et al., 2016b). Removing variables VIF >10 eliminated multicollinearity issues that might affect modeling. The least-correlated set of variables were used to re-run model using the above mentioned modeling parameters for host and pathogen. The calibrated model was then projected onto future (2050) climatic scenarios.

### Model Validation and Detect Change in Suitable Area of Tea Growing Areas

Model prediction performance was calculated using TSS (True Skill Statistics), MaxKappa, and AUC (Area Under Curve-Receiver Operating characteristics) statistics (Araujo et al., 2005). The calculated TSS is independent of prevalence—the ratio of presence to pseudo-absence data in the presence–absence predictions (Allouche et al., 2006). Both TSS and MaxKappa deal with sensitivity and specificity values of model output. Both range from −1 to +1, where +1 indicates perfect agreement and TSS >0.7 indicates a good model fit (Allouche et al., 2006). Both TSS and MaxKappa deal with sensitivity and specificity values of model output. Both range from −1 to +1, where +1 indicates perfect agreement and TSS >0.7 indicates good model fit (Allouche et al., 2006). The average model was converted to a binary model (presence/absence) applying equal training sensitivity and specificity thresholds that suit the present distribution of the focal species. Reclassified binary layers were used to estimate suitability change in the future for hosts and pathogens. We compared the model predictions in different time frames: baseline and projection in the future (2050) across four RCPs. The modeled occurrence probabilities for both current and future climatic conditions were summarized as presence–absence of tea distribution, and the overall distribution change was calculated as the difference between two distribution models for current and future conditions (Ranjitkar et al., 2016a; Ranjitkar et al., 2016b). A fuzzy logic model was employed for identifying areas where fungal pathogens overlap with tea-growing areas.

## Results

### Tea Distribution

**Figure 1:**
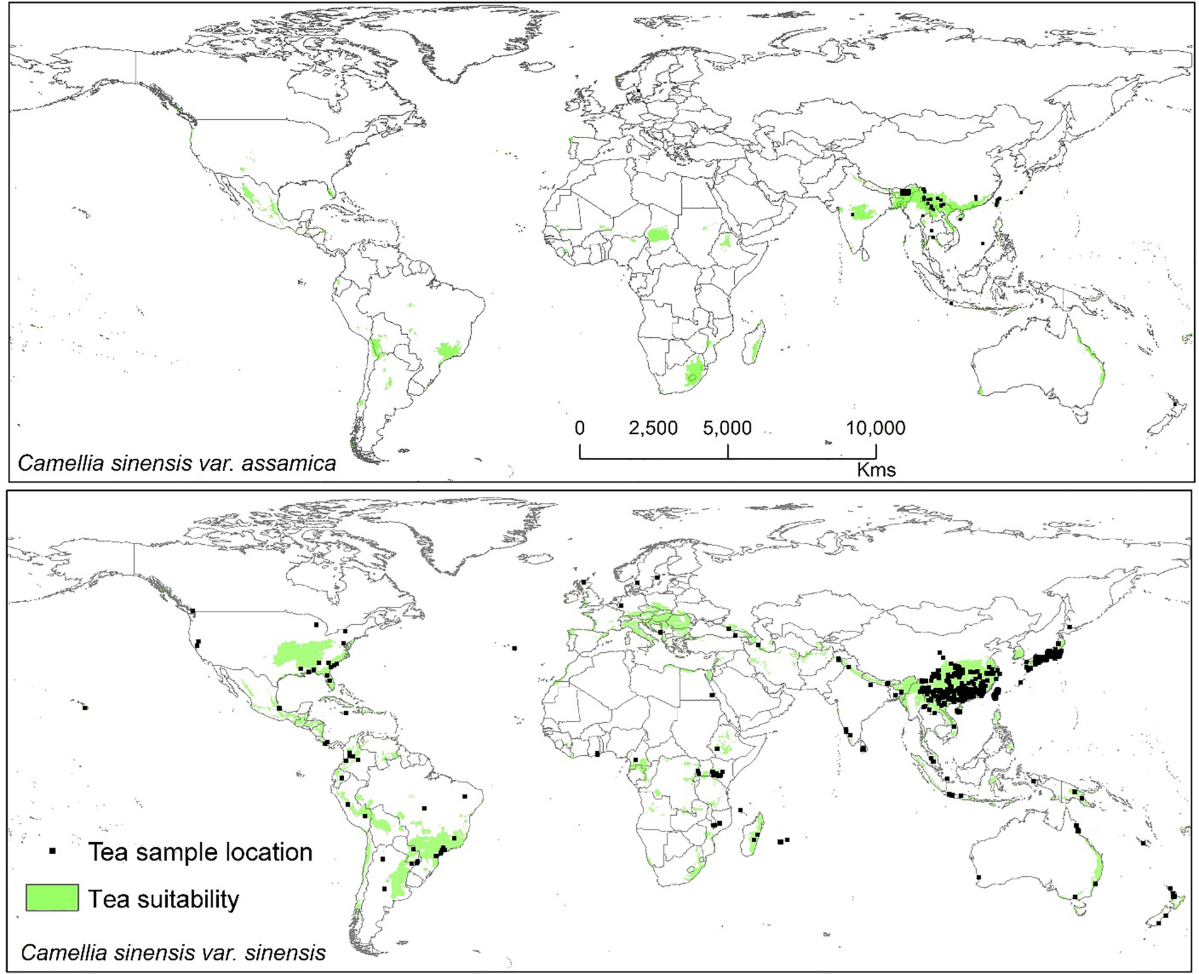
Suitable area for *C. sinensis* var. *sinensis* and *C. sinensis* var. *assamica* growing in baseline climatic conditions (green represents the suitable areas). Tea species occurrence records by different plant collectors and deposited in various herbaria across the world. Here locations of species occurrence records are plotted, and in the background tea-suitable area in baseline climatic scenario is shown.

**Figure 2:**
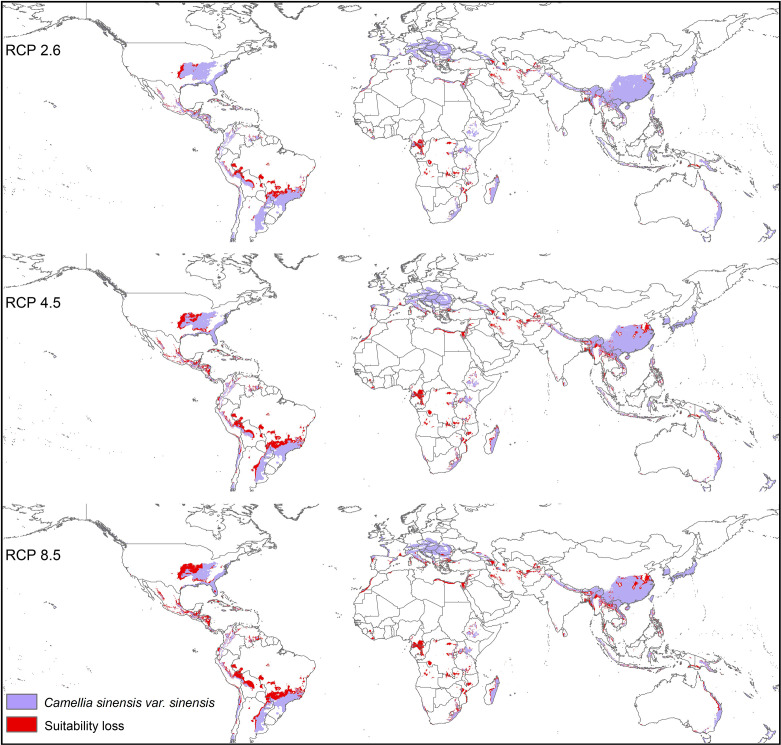
Suitable areas for *C. sinensis* var. *sinensis* growing under future climate scenarios with baseline suitable areas and losses by 2050 (purple represents the suitable areas, and red represents lost suitable areas).

**Figure 3:**
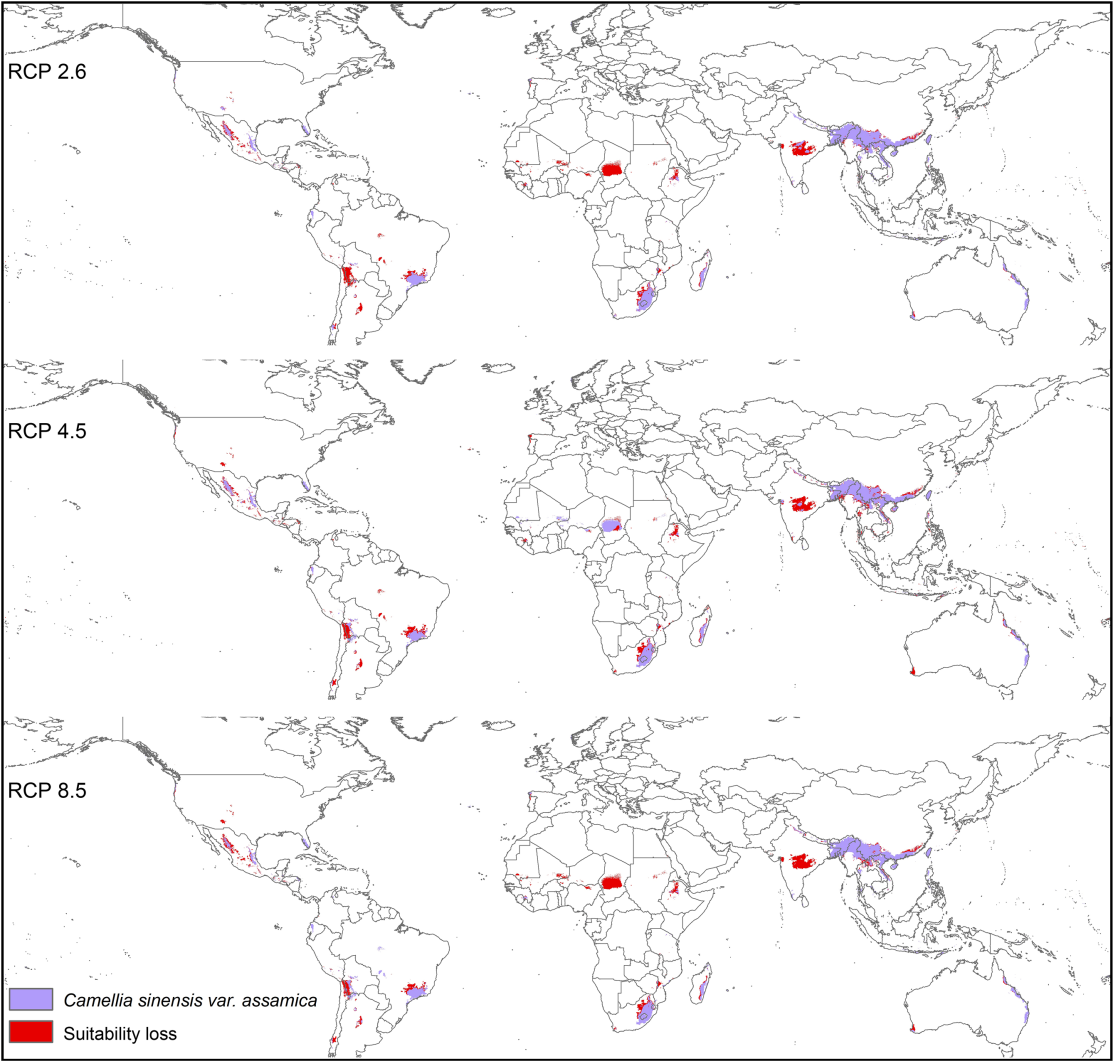
Suitable areas for *C. sinensis* var. *assamica* growing under future climate scenarios with baseline suitable areas and losses by 2050 (purple represents the suitable areas, and red represents lost suitable areas).

Modeled suitable habitats for both varieties of commercial tea under baseline and projected future climatic conditions are shown in **Figures 1**–**3**. Intentionally we did not include novel areas for tea plantations as those areas already had other land-use types, and converting to tea plantation will not be economically beneficial. This also refers to Ranjitkar et al. (2016a) who showed the evidence of the distribution of suitable habitats and impacts of climate change for the selected tree species. Tea species occurrence records of collection sites across the world are shown separately. Almost all tea species occurrence records are in the modeled suitable range of tea. Modeled area is largely represented where sample collection was larger and where sample size was small or just one to two projected grids are smaller. Sample size and representation from each geographical region affect final model output (**Figure 1**). The AUC values ranged from 0.97 to 0.99, TSS between 0.65 and 0.95, and maxKappa between 0.31 and 0.48 for all models. CSS has larger and more widely distributed habitats than CSA. The main areas suitable for CSS are forecasted across different countries in Asia, Africa, Europe, and North and South America (**Figure 1**). Commercial cultivation is just beginning in Europe and North American countries (Zhang et al., 2020). Suitable areas for tea growing under future climate scenarios will decrease in land coverage, especially in South and Southeast Asia, Central and South America, and Africa. Models revealed CSS might lose 15 to 32% of suitable area by 2050 compared to baseline. Predicted suitability loss was higher in RPC 8.5 scenario compared to RPC4.5 and least in RPC2.6 (**Figure 2**). Our modeling results revealed climatic suitability of CSA is distributed mainly across the countries in Asia, while suitable area is also predicted across African and South American countries (**Figure 1**). These suitable areas are forecast to decrease 32 to 34% by 2050. All scenarios, the minimum (RCP 2.6), the median (RCP4.5), and the maximum (RCP 8.5) climate change projections in 2050, suggested very similar trends in terms of the projected changes (**Figure 3**). Areas where major loss is detected in the model are highlighted in the inset in **Figures 2** and **3**. The contribution of each replicate to the final average model was proportional to their goodness-of-fit statistics.

### Climatic Factors *vs.* Tea Disease Development and Fungal Pathogen Modeling

**Figure 4:**
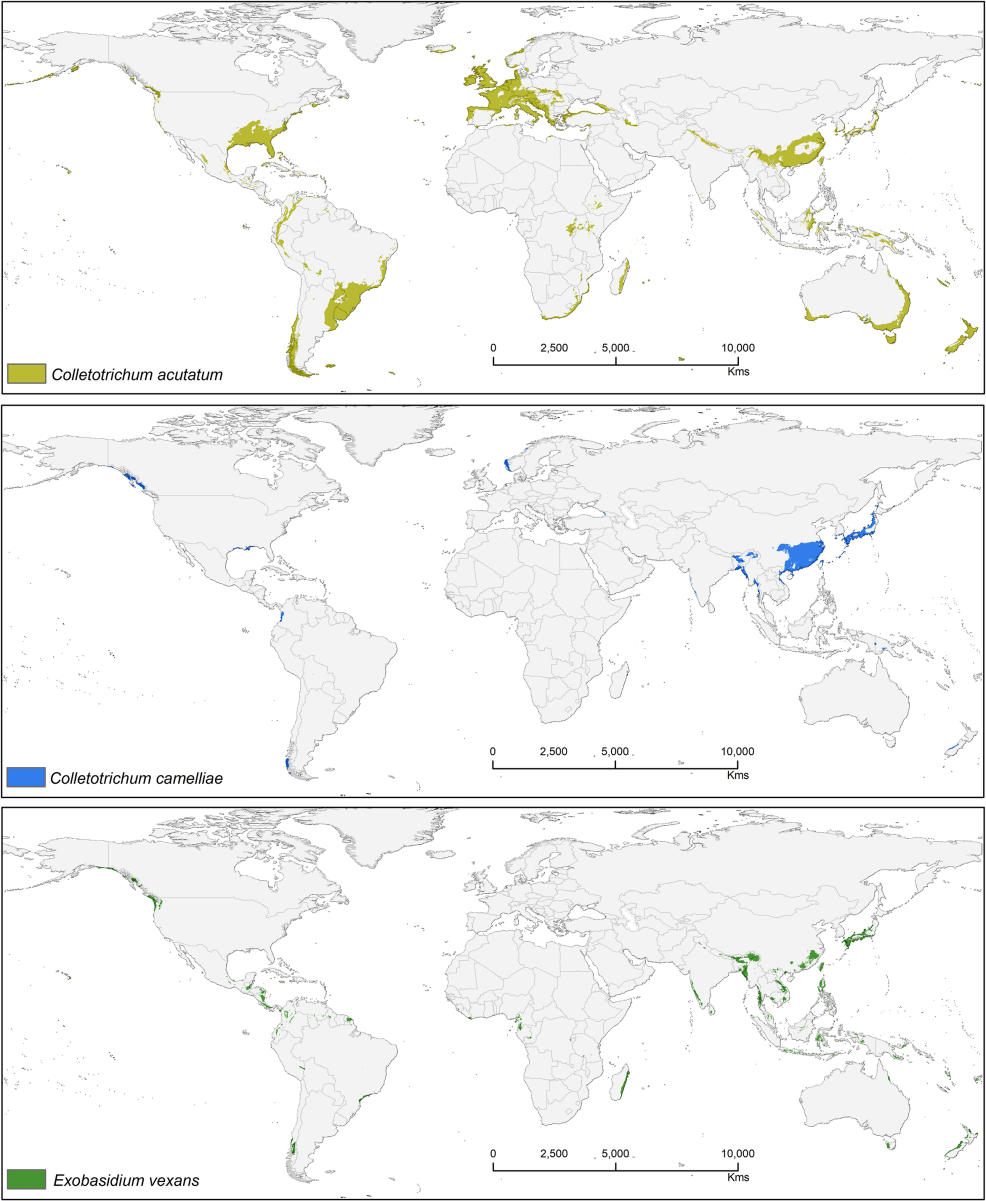
Bioclimatic suitability for three major fungal pathogens: *Colletotrichum acutatum, Colletotrichum camelliae*, and *Exobasidium vexans*.

**Figure 5:**
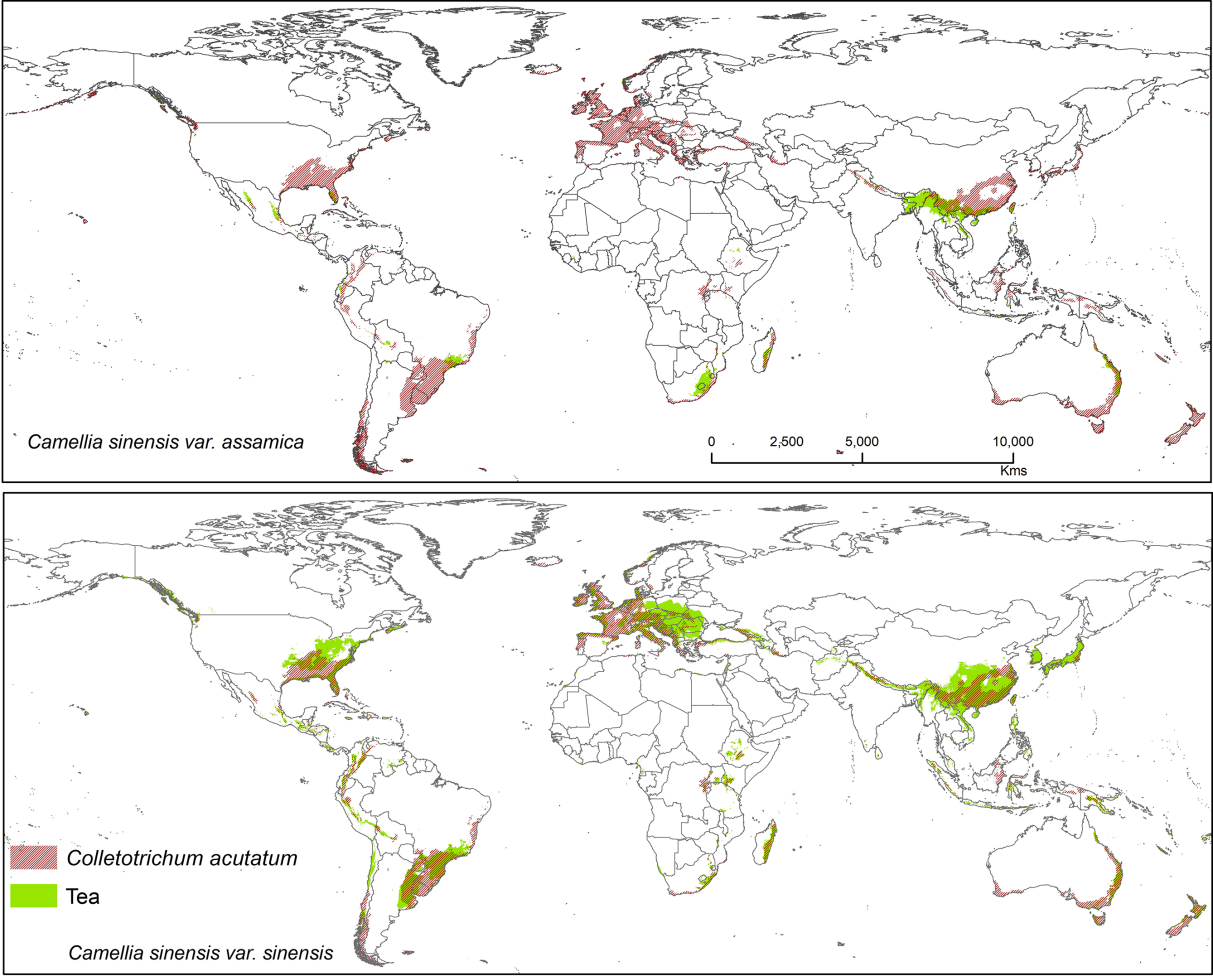
Shows the suitable area of *Colletotrichum acutatum* on tea with suitable area of two varieties of tea as projected in the average of all future projections.

**Figure 6:**
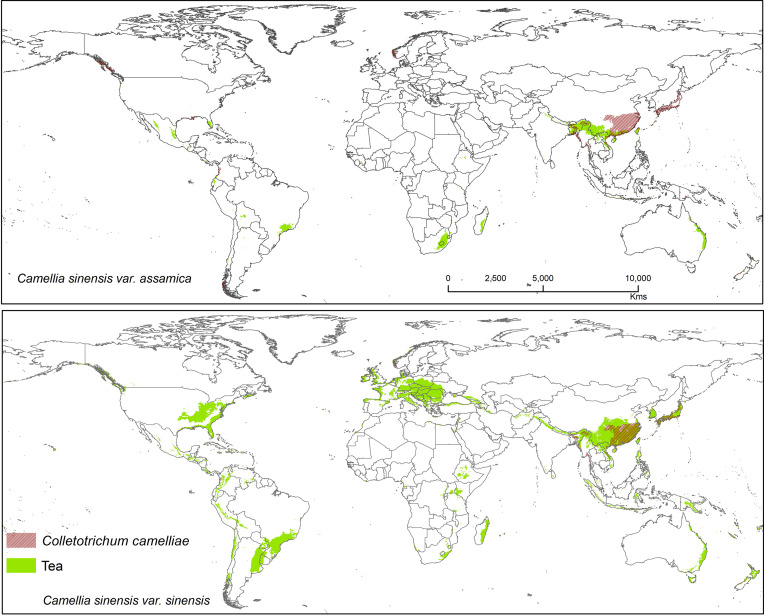
Shows the suitable area of *Colletotrichum camelliae* on tea with suitable area of two varieties of tea as projected in the average of all future projections.

**Figure 7:**
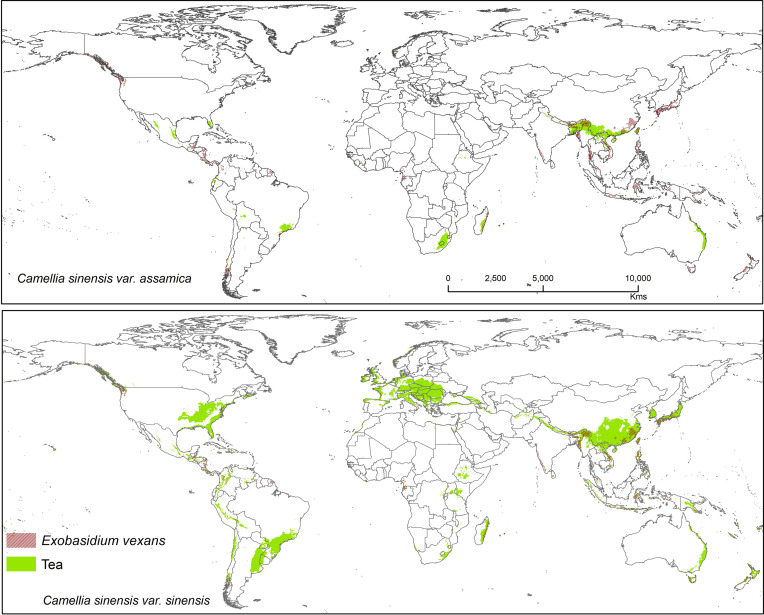
Shows the suitable area of *Exobasidium vexans* on tea with suitable area of two varieties of tea as projected in the average of all future projections.

Suitable area of each fungal pathogens on two varieties of tea show substantial overlap in **Figures 5**–**7**, while modeled tea and its fungal pathogens with overlapping suitability, unsuitability, new habitat, and pathogen free tea suitability are shown in **Supplementary Table S3**. The model analysis indicates in future climatic scenarios a suitable range of fungal species that overlap with suitable tea (respectively in CSS and CSA) growing areas are *Co. acutatum* (44.30%; 31.05%), *Co. camelliae* (13.10%; 10.70%), and *E. vexans* (10.20%; 11.90%). Not only tea plantations will suffer from those pathogens but other land-use types in Ranjitkar et al. (2016a) might also be affected. Modeling habitat suitability for tea fungal pathogens reveals that their distributions differ across spatial extent (**Figure 4**). However, these pathogens are widely co-distributed with tea (**Figures 5**–**7**).

#### Colletotrichum acutatum

*Colletotrichum acutatum* is an important anthracnose pathogen with a wide host plant range worldwide (Damm et al., 2012). On tea plants, this pathogen is considered a new causal agent which can reduce quality of tea leaves when compared with *Co. gloeosporioides*, as this species has high phenotypic and genotypic diversity (Chen et al., 2017). Brown blight on tea leaves caused by *Co. acutatum* was reported by Chen et al. (2016; 2017), showing *Co. acutatum* is a suitable candidate pathogen to study climatic change and disease development on tea. The *Co. acutatum* species complex is known today as a destructive pathogen on fruits (including perennial crops), such as citrus (Peres et al., 2008), apple (Lee et al., 2007), olive (Talhinhas et al., 2011), and blueberry (Wharton and Schilder, 2008). According to our prediction, it shares a high percentage of bioclimatic suitability with tea at 44.30% (CSS) and 31.05% (CSA). *Colletotrichum acutatum* is thus a suitable candidate pathogen on tea plants that is capable of shifting to novel areas or hosts under climate change scenarios. Suitable modeled areas for *Co. acutatum* on CSA include southern China, North India, and Nepal, while suitable areas for CSS include Asia (Southern China, Japan, North India, and Nepal), Africa (Madagascar), Europe (Central and Wester), North America (United States), and South America (Southern Brazil, Uruguay, Argentina, Colombia, Peru, and Chile) (**Figure 5**).

#### Colletotrichum camelliae

*Colletotrichum camelliae* has been reported among the most prevalent tea pathogens causing damage and several diseases known as tea leaf blight, tea brown blight, or tea anthracnose (De Silva et al., 1992; Wang L. et al., 2016; Wang Y. et al., 2016; Ponmurugan et al., 2019). Orrock et al. (2020) reported that anthracnose in US tea is caused by this fungus. *Colletotrichum camelliae* is the most dominant and frequently identified pathogen in the main tea growing areas of China (Liu et al., 2015; Wang L. et al., 2016). This species is likely still a dominant pathogen on tea, especially in Asia where it affects CSS.Tea in China is likely to be further affected by this pathogen under future climates because in China it was shown that the frequency of extreme weather and climate events will be increasing (Baby et al., 1998). Suitable modeled areas for *Co. camelliae* on tea plants are found in Asia with a bioclimatic suitability percentage of 13.10% (CSS) and 10.70% (CSA) (**Figure 6**). *Colletotrichum* spp. pathogens show the way in which the growth rate of spore germination and onset of diseases vary in accordance to temperature change, with the temperatures >25 and <35°C representing ideal conditions for fungal growth. Tea-growing areas in most places might become favorable climatic zones for pathogens, and in these areas fungal infections in tea might become widespread. T1 = 25°C and T2 = 27.5°C are temperatures at which germination and appressorium were at their peak (**Supplementary Figure S1**). This information could foster increased human preparedness in the emergence of new latent pathogen with climate change. The results show that temperatures between 25 and 30°C foster ideal conditions for this kind of fungal growth (**Supplementary Figure S1**). Our results are in accordance with Piao et al. (2010) who commented that when rainfall or high humidity accompanies a temperature range between 23 and 35°C, the disease may become most widespread and serious. According to available literature, 25 to 28°C is the optimum range of temperatures and is justified for the selected temperature range (Pasanen et al., 1991; Pietikäinen et al., 2005; Piao et al., 2010). The germination time and growth rate of a fungus depend on temperature, humidity (50–70%), and surface nutrients (Pasanen et al., 1991; Piao et al., 2010).

#### Exobasidium vexans

*Exobasidium vexans* is an obligate parasite or endemic pathogen which occurs only in tea plant and causes blister blight disease on tea leaf and is the most devastating disease that affects tea, especially in Asia (south and east asia) (Punyasiri et al., 2005; EPPO, 2006; Sinniah et al., 2016; Ponmurugan et al., 2019; Chalkley, 2021). This pathogen has caused severe crop/yield losses in Sri Lanka (33%), India (35–50%), and Indonesia (20–25%) (De Silva et al., 1992; Baby et al., 1998; Radhakrishnan and Baby, 2004; Basu-Majumder et al., 2010; Diez et al., 2013). This fungus is known to remain dormant during periods of unfavorable conditions, only becoming pathogenic under optimal climatic conditions. If not controlled by fungicides, tea losses due to this pathogen may range from 25 to 43% (Mur et al., 2015). Suitable modeled areas for *E. vexans* on tea are in Australia, South America, Africa, and Asia. In future, CSS maps have more overlab with pathogen maps than CSA (**Figure 7**). It has a bioclimatic suitability percentage with suitable tea of 10.20% (CSS) and 11.90% (CSA) and areas with increased future risk cluster in Asia, including China, Japan, Mynmar, Vietnam, India, Bhutan, Sri Lanka, Philippines, and Indonesia (**Figure 7**).

Correspondence analysis among the three tea pathogens used in our study with global annual temperatures, annual precipitation, and humdity indicates that these pathogens might have the ability to survive low humidity and temperatures under 15°C (**Supplementary Figures S2**–**S5**). These diseases are thus able to preceed tea into novel areas as emergent pathogens, potentially impacting new tea production initiatives in the future.

## Discussion

The effects of crop losses associated with pathogens and pests in major agricultural crops are better understood than those of commodity crops like tea (Chen et al., 2019; Savary et al., 2019), and among perennial crops tea is unique as its leaves are the product. We modeled tea growth under future climate scenarios and found that suitable tea-growing areas could decrease 34% by 2050. In addition, fungal pathogenic mapping revealed an overlap with baseline tea-growing areas. We anticipate that climate change will render large areas unsuitable for the two dominant tea varieties, CSS and CSA. Future pathogen damage will likely be greater in growing areas for the *sinensis* variety as *C. sinensis* grows in relatively cooler areas compared to *C. assamica*, and most of the pathogens are active in these regions compared to warmer areas where *C. assamica* grows. In addition to change in suitability, temperature rise in baseline tea-plantation areas favors rapid growth and spread of fungal pathogens. We observed that future temperatures will reach optimum levels for these selected pathogens in baseline tea-plantation areas. Suitable areas of overlap for both hosts and pathogens were estimated to show potentially threatened areas (**Figures 5** **–** **7**). Furthermore, potential changes in the distribution were assessed by comparing the suitability map produced by SDM under future climates.

Tea growing areas comprise regions with diverse climates. Although climate change affects each region differently, it influences tea yields by altering precipitation levels, increasing temperatures, and encouraging pests. Bunn et al. (2015) reported that climate change will reduce the global area suitable for coffee by about 50% across emission scenarios. Compared to coffee (Bunn et al., 2015), our model for tea revealed reduction of global area suitable for tea will be less. Still, climate change will exert an enormous influence on future tea production. This future projection information identifies overlapping regions of fungal species with suitable tea while, overlapping regions potential for other plantations as well. Similarly, the impact of climate change is also reported on several agroforestry tree species in Yunnan Province, China, and it showed a decline from 7 to 18% (Ranjitkar et al., 2016a), also vegetation shifts, expansion of alpine vegetation zones (Brandt et al., 2013), and a decrease of *Abies georgei* forests under different emission scenarios between 4.6 and 25.9% in the elevational range can be seen (Wong et al., 2010). According to Orrock et al. (2020), recommendations to focus on tea research for development, such as test accessions, vary responses to the disease, applying the methods developed here to identify disease tolerant and future breeding efforts towards developing cultivars for tea production. Species distribution or niche models, such as Maxent, use the tolerance limit to critical factors based in the current species distribution and the climatic or other factors we input to the model. The predicted species distribution is a theoretical or the fundamental niche based on the input climatic factors, and there are other factors that might affect the actual distribution of species (Elith and Franklin, 2013; Seybold et al., 2020). In the current study, we use two approaches, the tea plant distribution based on climatic factors and the distribution of known pathogens in the same climatic scenario, which we believe is an advancement in the estimate of species distribution. However, there are other factors that affect the actual niche; for example, with the change of climatic conditions, the susceptibility of tea for the mentioned pathogen might also change (Toyoda et al., 2002). Modeling other factors, especially complex biotic factors, such as susceptibility to pathogens, chemical or mechanical herbivore defense, should be the concerns of future research, and the current result should be interpreted with caution.

In addition to change in suitability, temperature increases in baseline tea-plantation areas will favor rapid growth and the spread of fungal pathogens. We found that future temperatures will reach optimum levels for these selected pathogens in baseline tea-plantation areas. This also refers to plant natural microbiota interaction with new pathogens which underscores the need to reduce the lag time between the appearance of new diseases and development of protective measures effective on a broad range of pathogens and host plants.

We are just considering 30 to 50 years of change, and within this time period genetic mutation in higher plants such as tea is not possible. However, plasticity can be considered. Due to phenotypic plasticity plants can adapt to a wide range of climates and survive in adverse conditions.

One major limiting factor of this study is the limited knowledge of tea–pathogen distributions; however, this does not imply that pathogens are absent from non-reporting regions. Climate factors other than temperature and humidity (extreme events, CO_2_ fertilization, ozone, nitrogen deposition, and drought conditions) will also change, affecting tea and its interactions with pathogens. Stress-induced changes in tea from climate change have been explored by Han et al. (2018) *via* physiology, biochemistry, metabolism, and the economic and societal aspects of tea under changing climatic conditions. In addition, the identified fungi pathogens based on blast searches or only ITS sequences are unacceptable for species level because of the intraspecific variations when only one gene is used for phylogenetic analysis specially the genus *Colletotrichum* (Shi et al., 2018; He et al., 2020).

The effects of climate and pathogens on tea can be addressed in several ways. Adding shade tree species to tea plantations may mitigate damages from epidemic pests and diseases (Mukhopadhyay and Mondal, 2017). Increased knowledge of fungal pathogen genomes will facilitate the production of new fungicides (Cools and Hammond-Kosack, 2013). Understanding fungal communities or fungal pathogen–plant interactions by using next-generation sequencing, genomics, and metabolomics will help to uncover fungal infection mechanisms and plant defense mechanisms, which could be helpful in finding new targets for fungicide development and finding useful resistant genes for crop breeding (Pasiecznik et al., 2005; James et al., 2006; Peay et al., 2016; Chen et al., 2019b). Even though a number of studies have been conducted on identification of fungal pathogens on tea, only a few detailed studies have been done on fungal pathogen genomics on tea. Metagenomics have many advantages on pathogen evolution, host–pathogen interactions, determination of trait-specific genes and plant–host adaptation mechanisms (Li et al., 2016; Ren et al., 2019). Ultimately, understanding the roles of pathogens in crop production systems might even enhance food security (Gregory et al., 2009). Currently, tea plants are mostly protected from fungal diseases by fungicides, but these come with attendant harmful side effects on the tea plant, human health, the environment, and biodiversity. Fungi are distributed widely and are able to colonize and infect all plant tissues, as they have adapted many different evolutionary strategies, and several of them are able to switch modes from non-pathogenic to pathogenic (James et al., 2006; Möller and Stukenbrock, 2017). Finally, more attention is needed to understand the antagonism of endophytic fungi, such as that found in the genus *Diaporthe*, which is known as the most abundant genus of endophytes in tea plants and is associated with the symptomatic and asymptomatic tissues of *Camellia* spp. from several provinces in China (Chen, 2007; Osono and Hirose, 2009; Gao et al., 2016). Rising temperatures in tea plantations will increase their suitability for pathogens, which increase probability of pathogen dispersal and development, thus increasing the probability of future crop damage in 2050. For example, *Diaporthe theae* causes a common disease (canker disease), which creates serious problems in all tea-growing regions of the world (Ponmurugan et al., 2006). Under climate change, this species is able to affect tea leaves, leading to severe economic losses (Guarnaccia and Crous, 2017; Wang et al., 2017). Moreover, the genus *Pestalotiopsis* (including *Pestalotiopsis*-like species) needs to be considered as they also can cause several diseases on the foliage, stems, and roots of tea plant; of the *Pestalotiopsis/Pestalotiopsis*-like species, *Pestalotiopsis camelliae*, *Pseudopestalotiopsis theae*, and *Pseudopestalotiopsis camelliae-sinensis* are considered global tea pathogens (Joshi et al., 2009; Maharachchikumbura et al., 2011; Zhang et al., 2012; Maharachchikumbura et al., 2013; Liu et al., 2017; Chen et al., 2018) capable of causing substantial tea production losses. If there is greater variation in temperature (lower as well as higher), this disease is likely to become more serious.

This study provides an understanding of pathogens on tea, and these results will facilitate further research on the potential risks of fungal pathogens and how to prepare for risks in advance. Other perennial crops will also be challenged by new climates and pathogen interactions, also requiring additional study.

## Data Availability Statement

The original contributions presented in the study are included in the article/**Supplementary Material**. Further inquiries can be directed to the corresponding authors.

## Author Contributions

PM, SR, JX, YD, and JS made significant contributions to design the study. ST, SK, SR, and DS wrote the manuscript. SR provided the models and maps. KH, RJ, IM, and DB verified the methodology and modeling as well as providing valuable comments and suggestions. All authors contributed to the article and approved the submitted version.

## Funding

This work was supported by The Key Research Project, Agroforestry Systems for Restoration and Bio-industry Technology Development (Grant No. 2017YFC0505101), Ministry of Sciences and Technology of China (Grant No. 2017YFC0505100), the Yunnan Provincial Key Programs of Yunnan Eco-Friendly Food International Cooperation Research Center project (2019ZG00908), China Postdoctoral Science Foundation, the Yunnan Human Resources and Social Security Department Foundation, and ChiangMai University.

## Conflict of Interest

Author SR was employed by N. Gene Solution of Natural Innovation, Kathmandu, Nepal.

The remaining authors declare that the research was conducted in the absence of any commercial or financial relationships that could be construed as a potential conflict of interest.

## Supplementary Material

Supplementary Table 1 Global perennial crop markets ($USD Billions per year).

Supplementary Table 2 Fungal diseases reported from Camellia sinensis, confirmed by morphology, genomics and pathogenicity (the bold type shows pathogens used in this study).

Supplementary Table 3 Modeled tea and its fungal pathogens overlapping suitability and pathogen free tea suitability

Supplementary Figure 1 Temperature induced fungal pathogen growth with percent germination, appressorium and anthracnose disease. Temperature 25°C or above but below 30°C are better conditions for fungal growth, in that range, tea might be severely infected causing major economic loss (Kenny et al., 2012).

Supplementary Figure 2 Temperature ranges for tea and its pathogens based on occurrence points.

Supplementary Figure 3 Average annual temperature map of the world. Baseline tea plantations and selected pathogen occurrence areas.

Supplementary Figure 4 Precipitation ranges for tea and its pathogens.

Supplementary Figure 5 Annual relative humidity map of the world. Baseline tea plantations and selected pathogen occurrence areas.
