# Supplementary material for: Climate-Fungal Pathogen Modeling Predicts Loss of Up to One-Third of Tea Growing Areas
Source: Front Cell Infect Microbiol. 2021 Apr 29;11:610567. doi: 10.3389/fcimb.2021.610567 (PMC8116803; doi:10.3389/fcimb.2021.610567)
Supplement: Supplementary Table 1 — Global perennial crop markets ($USD Billions per year). [file DataSheet_1.docx]

Supplementary Material

# Supplementary Figures and Tables

## Supplementary tables

**Supplementary table S1 |** Global perennial crop markets ($USD Billions per year).

| Global perennial crop | USD Billions/year | References |
| --- | --- | --- |
| Coffee | 42 | Gaille, (2017) |
| Tea | 50 | Bedford, (2020) |
| Tree fruits | 72 | Van Rijswick, (2018) |
| Tree nuts | 62 | Market research future, (2020) |
| Total | **226** |  |
| Tea % of total | **22%** |  |

**Supplementary table S2 |** Fungal diseases reported from *Camellia sinensis*, confirmed by morphology, genomics and pathogenicity (the bold type shows pathogens used in this study).

| **Fungal disease** | **Pathogen/s** | **Host and distribution as mentioned in publications** | **Notes** | **References** |
| --- | --- | --- | --- | --- |
| Anthracnose | *Alternaria alternata* | *C. sinensis* (from tea trees grown in Luotian, Hubei, China) | ND^#^ | Zhou and Xu 2014** |
|  | *Colletotrichum aenigma* | *C. sinensis* (from the main tea growing regions in China) | *C. sinensis* var. *sinensis* | Wang et al., 2016a, b |
|  | ***Colletotrichum camelliae*** | **-*C. sinensis* (from six different commercially available tea, US)**  **-*C. sinensis* (from the main tea growing regions in China)** | ***C. sinensis* var. *sinensis*** | Wang et al., 2016a, b; Orrock et al., 2020 |
|  | *Colletotrichum endophyticum* | *C. sinensis* (from the main tea growing regions in China) | ND^#^ | Wang et al., 2016a, b |
|  | *Colletotrichum fructicola* | *C. sinensis* (from tea plants in growing regions of Jiaoling, Guangdong, China) | ND^#^ | Shi et al., 2017** |
| Brown-black | *Pestalotiopsis theae* (current name=*Pseudopestalotiopsis theae*) | *C. sinensis* (from main tea cultivation region of Hubai, China) | ND^#^ | Wang et al., 2017 |
| Brown blight | ***Colletotrichum acutatum*** | ***C. sinensis* (from popular tea cultivars in commercial tea estates of Wansheng, Chongqing, China)** | **ND^#^** | Chen et al., 2016**, 2017 |
|  | *Colletotrichum gloeosporioides* | *C. sinensis* (from Yellow Mountain fuzz tip, Anhui, China) | ND^#^ | Chen et al., 2017b; Guo et al., 2014** |
| Collar canker or dieback | *Ceratocystis fimbriata* | *C. sinensis* var. *assamica* (from Pu’er tea plant, Yunnan, China) | *C. sinensis*var. *assamica* | Xu et al., 2019** |
|  | *Fusarium solani* (current name=*Neocosmospora solani*) | *C. sinensis* (from tea fields in Sri Lanka) | *C. sinensis* var.*lasiocalyx* | Sinniah et al., 2017 |
| Foliar fungal | *Alternaria alternata* | *C. sinensis* (from nursery-grown tea plants, Dooars, North Bengal, India) | ND^#^ | Chakraborty et al., 2006** |
| Grey blight | *Neopestalotiopsis clavispora* | *C. sinensis* (from several commercial tea plantations, Chongqing, China) | ND^#^ | Chen et al., 2018 |
|  | *Pestalotiopsis camelliae* | *C. sinensis* (from tea plantations, Chongqing, China) | ND^#^ | Chen et al., 2017a**, 2018 |
|  | *Pestalotiopsis lushanensis* | *C. sinensis* (from tea plantations of Yongchuan, Chongqing China) | ND^#^ | Mur et al., 2015 |
|  | *Pseudopestalotiopsis camelliae-sinensis* | -*C. sinensis* (from several commercial tea plantations, Chongqing, China)  -*C. sinensis* (from primary tea-producing provinces, China) | ND^#^ | Chen et al., 2018 |
| Leaf blight/Blister blight | ***Exobasidium vexans*** | ***C. sinensis* (from tea plants, North Indian Assam variety growing within Margaret's Hope Tea Gardens (North Kurseong, Darjeeling, India))** | **“it merely reflects the fact that no one has undertaken a thorough scientific assessment of these tea varieties”** | Mur et al., 2015 |
|  | *Nigrospora sphaerica* (current name=*Nigrospora oryzae*) | *-C. sinensis* (from Qingyang, Anhui, China)  -*C. sinensis* (from commercial tea estates of the Darjeeling district, India) | ND^#^ | Dutta et al., 2015**; Liu et al., 2016** |
| Leaf necrosis/leaf spots | *Alternaria alternata* | *C. sinensis* (from tea trees, Luotian, Hubei, China) | ND^#^ | Zhou and Xu 2014** |
|  | *Botryosphaeria dothidea* | *C. sinensis* (from different farms of different counties, Fujian, China) | *C. sinensis* var. *sinensis* | Jayawardena et al., 2016 |
|  | *Epicoccum sorghinum* | *C. sinensis* (from Dushan, Guizhou, China) | ND^#^ | Bao et al., 2019 |
|  | *Lasiodiplodia pseudotheobromae* | *C. sinensis* (from fields in Zhangzhou, Fujian, China) | ND^#^ | Li et al., 2019a, b |
|  | *Lasiodiplodia theobromae* | *C. sinensis* (from fields in Zhangzhou, Fujian, China) | ND^#^ | Li et al., 2019a, b |
|  | *Phoma herbarum* | *C. sinensis* (from tea plant in Chizhou, Anhui, China) | ND^#^ | Thangaraj et al., 2018 |
|  | *Phyllosticta capitalensis* | *C. sinensis* (from tea plant in Chizhou, Anhui, China) | ND^#^ | Cheng et al., 2019 |

ND^#^ = No data or no responses from authors of the original study but crossed check with our data and those pathogens overlapped with our tea distribution map (**Figures 1-3**).

**=confirmed by blast searches, not based on types and may be wrong.

**Supplementary table S3 |** Modeled tea and its fungal pathogens overlapping suitability and pathogen free tea suitability

| Species | Overlapping suitability (number of grids) | Pathogen free tea suitability  (number of grids) | Percent | Remark |
| --- | --- | --- | --- | --- |
| *Camellia sinensis var. sinensis* | | | | |
| *C. sinensis var. sinensis* | 191,916 |  |  |  |
| *Co. acutatum* | 85,169 | 106,747 | 44.38% | Overlap to tea |
| *Co. acutatum* |  |  | 55.62% | Pathogen free |
| *Co. camelliae* | 25,245 | 166,671 | 13.15% | Overlap to tea |
| *Co. camelliae* |  |  | 86.85% | Pathogen free |
| *E. vexans* | 19,526 | 172,390 | 10.17% | Overlap to tea |
| *E. vexans* |  |  | 89.83% | Pathogen free |
| *Camellia sinensis var. assamica* | | | | |
| *C. sinensis var. assamica* | 50,958 |  |  |  |
| *Co. acutatum* | 15,822 | 35,136 | 31.05% | Overlap to tea |
| *Co. acutatum* |  |  | 68.95% | Pathogen free |
| *Co. camelliae* | 5,464 | 45,494 | 10.72% | Overlap to tea |
| *Co. camelliae* |  |  | 89.28% | Pathogen free |
| *E. vexan* | 6,082 | 44,876 | 11.94% | Overlap to tea |
| *E. vexan* |  |  | 88.06% | Pathogen free |

## Supplementary figures


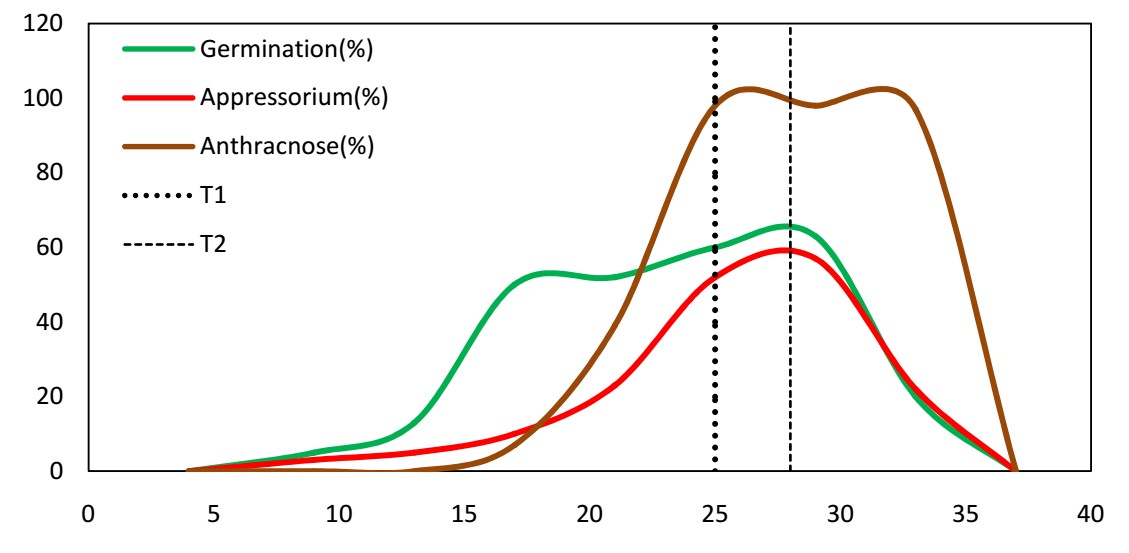


**Supplementary figure S1 |** Temperature induced fungal pathogen growth with percent germination, appressorium and anthracnose disease. Temperature 25 °C or above but below 30 °C are better conditions for fungal growth, in that range, tea might be severely infected causing major economic loss (Kenny et al., 2012).


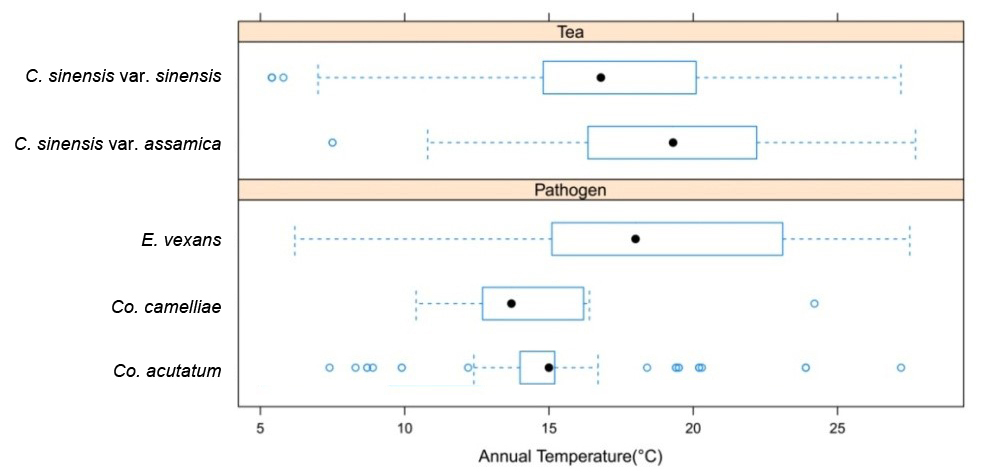


**Supplementary figure S2 |** Temperature ranges for tea and its pathogens based on occurrence points.


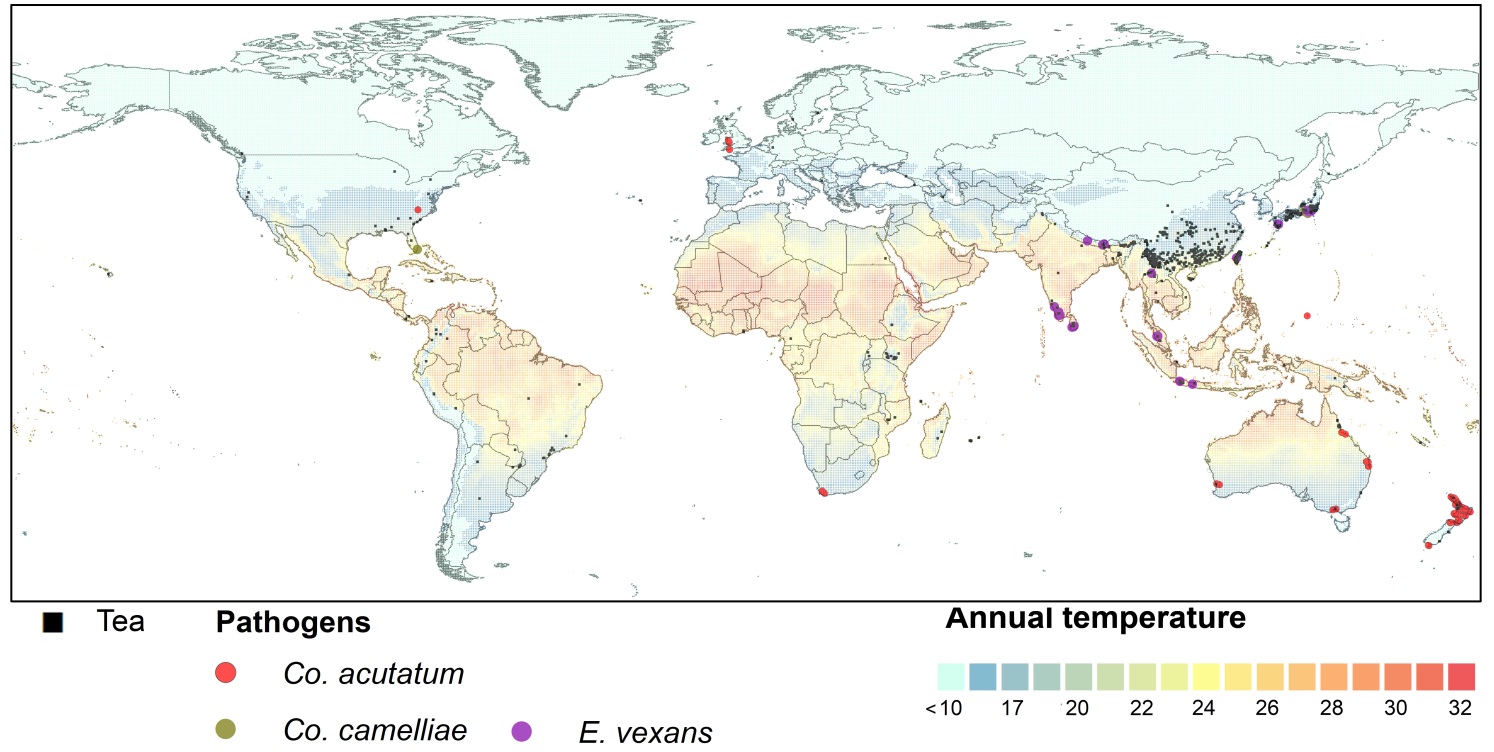


**Supplementary figure S3 |** Average annual temperature map of the world. Baseline tea plantations and selected pathogen occurrence areas.


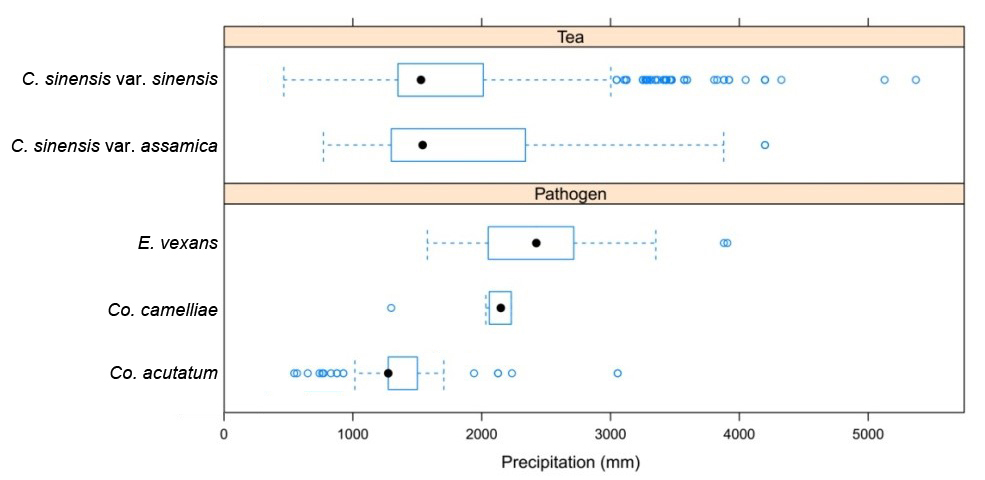


**Supplementary figure S4 |** Precipitation ranges for tea and its pathogens.


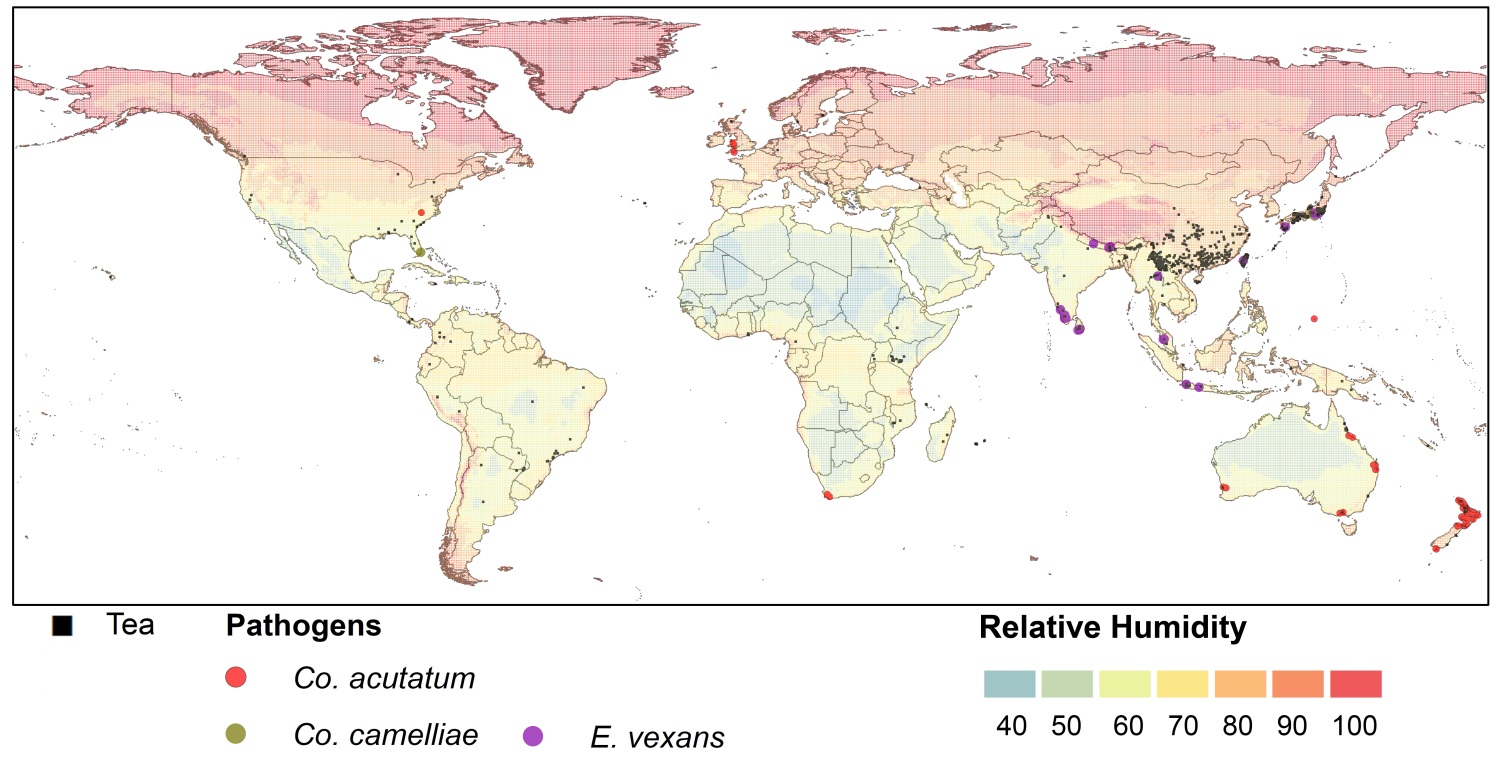


**Supplementary figure S5 |** Annual relative humidity map of the world. Baseline tea plantations and selected pathogen occurrence areas.

**References**

Bao, X.T., Dharmasena, D., Li, D.X., Wang, X., Jiang, S.L., Ren, Y.F., et al. (2019). First report of *Epicoccum sorghinum* causing leaf spot on tea in China. *Plant Dis* 103, 3282. doi:10.1094/pdis-06-19-1296-pdn

Bedford, E. (2020). Global tea market size 2018-2026. Retrieved from https://www.statista.com/statistics/326384/global-tea-beverage-market-size

Chakraborty, B., Das-Biswas, R., and Sharma, M. (2006). *Alternaria alternata* - a new foliar fungal pathogen of tea in north Bengal, India. *Plant Pathol* 55, 303. doi:10.1111/j.1365-3059.2006.01330.x

Chen, Y., Qiao, W., Zeng, L., Shen, D., Liu, Z., Wang, X., and Tong, H. (2017b). Characterization, pathogenicity, and phylogenetic analyses of *Colletotrichum* species associated with brown blight disease on *Camellia sinensis* in China. *Plant Dis* 101, 1022-1028. doi:10.1094/PDIS-12-16-1824-RE

Chen, Y., Tong, H., Wei, X., and Yuan, L.Y. (2016). First report of brown blight disease on *Camellia sinensis* caused by *Colletotrichum acutatum* in China. *Plant Dis* 100, 227. doi:10.1094/PDIS-07-15-0762-PDN

Chen, Y., Zeng, L., Shu, N., Jiang, M., Wang, H.T., Huang, Y., and Tong, H. (2018). *Pestalotiopsis*-Like species causing gray blight disease on *Camellia sinensis* in China. *Plant Dis* 102, 98-106. doi:10.1094/PDIS-05-17-0642-RE

Chen, Y., Zeng, L., Shu, N., Wang, H., and Tong, H. (2017a). First report of *Pestalotiopsis camelliae* causing grey blight disease on *Camellia sinensis* in China. *Plant Dis* 101, 1034-1034. doi:10.1094/PDIS-01-17-0033-PDN

Cheng, L., Thangaraj, K., Deng, C., Deng, W., and Zhang, Z. (2019). *Phyllosticta capitalensis* Causes Leaf Spot on Tea Plant (*Camellia sinensis*) in China. *Plant Dis* 103, 2964. doi:10.1094/pdis-04-19-0768-pdn

Dutta, J., Gupta, S., Thakur, D., and Handique, P.J. (2015). First report of *Nigrospora* leaf blight on tea caused by *Nigrospora sphaerica* in India. *Plant Dis* 99(3), 417. doi:10.1094/PDIS-05-14-0545-PDN

Gaille, B. (2017). 17 Coffee consumption and industry sales statistics. Retrieved from https://brandongaille.com/16-coffee-consumption-and-industry-sales-statistics

Gao, Y., Liu, F., and Cai, L. (2016). Unravelling *Diaporthe* species associated with *Camellia*. *System. Biodivers* 14, 102-117. doi:10.1080/14772000.2015.1101027

Jayawardena, R.S., Li, X., Xu, W., Yan, J., Li, H.L., and Hyde, K.D. (2016). First report of *Botryosphaeria dothidea* causing leaf necrosis of *Camellia sinensis* in Fujian Province, China. *Plant Dis* 100, 854-854. doi:10.1094/PDIS-08-15-0930-PDN

Li, D., Bao, X., Ren, Y., Song, B., Chen, Z., and Wang, Y. (2019a). First report of *Lasiodiplodia theobromae* causing leaf spot on tea plant in Guizhou Province of China. *Plant Dis* 103, 374-374. doi:10.1094/pdis-06-18-1032-pdn

Li, H., Jayawardena, R.S., Xu, W., Hu, M., Li, X., Liu, J., Hyde, K.D., and Yan, J. (2019b). *Lasiodiplodia theobromae* and *L. pseudotheobromae* causing leaf necrosis on *Camellia sinensis* in Fujian Province, China. *Can. J. Plant Pathol* 41, 277-284. doi:10.1080/07060661.2019.1569559

Liu, Y.J., Tang, Q.H., and Fang, L.Y. (2016). First report of *Nigrospora sphaerica* causing leaf blight on *Camellia sinensis* in China. *Plant Dis* 100, 221-222. doi:10.1094/PDIS-04-15-0493-PDN

Market research future (2020). Retrieved from https://www.marketresearchfuture.com/press-release/global-tree-nuts-marketmarketresearchfuture

Mur, L.A., Hauck, B.B., Winters, A.L., Heald, J.K., Lloyd, A.J., Chakraborty, U., and Chakraborty, B. (2015). The development of tea blister caused by *Exobasidium vexans* in tea (*Camellia sinensis*) correlates with the reduced accumulation of some antimicrobial metabolites and the defence signals salicylic and jasmonic acids. *Plant Pathology* 64(6), 1471-1483. doi:10.1111/ppa.12364

Orrock, J.M., Rathinasabapathi, B., and Richter, B.S. (2020). Anthracnose in U.S. tea: pathogen characterization and susceptibility among six tea accessions. *Plant Dis* PDIS07191518RE. doi:10.1094/pdis-07-19-1518-re

Shi, N., Du, Y., Ruan, H., Yang, X., Dai, Y., Gan, L., and Chen, F. (2018). First report of *Colletotrichum fructicola* causing anthracnose on *Camellia sinensis* in Guangdong Province, China. *Plant Dis* 102, 241. doi:10.1094/PDIS-05-17-0705-PDN

Sinniah, G.D., Munasinghe, C.E., Mahadevan, N., Jayasinghe, S.K., and Kulatunga, D.C. (2017). Recent incidence of collar canker and dieback of tea (*Camellia sinensis*) caused by *Fusarium solani* species complex in Sri Lanka. *Australas. Plant Dis. Notes* 12, 1-6. doi:10.1007/s13314-017-0262-5

Thangaraj, K., Deng, C., Cheng, L., Deng, W., and Zhang, Z. (2018). Report of *Phoma herbarum* causing leaf spot disease of *Camellia sinensis* in China. *Plant Dis* 102, 2373. doi:10.1094/PDIS-01-18-0121-PDN

Van Rijswick, C. (2018). World fruit map 2018: global trade still fruitful. Retrieved from https://research.rabobank.com/far/en/sectors/regional-food-agri/world_fruit_map_2018.html

Wang, L., Wang, Y., Cao, H., Hao, X., Zeng, J., Yang, Y., and Wang, X. (2016b). Transcriptome analysis of an anthracnose-resistant tea plant cultivar reveals genes associated with resistance to *Colletotrichum camelliae*. *PloS One* 11. doi:10.1371/journal.pone.0148535

Wang, Y., Hao, X., Wang, L., Xiao, B., Wang, X., and Yang, Y. (2016a). Diverse *Colletotrichum* species cause anthracnose of tea plants (*Camellia sinensis* (L.) O. Kuntze) in China. *Scientific Reports* 6. doi:10.1038/srep35287

Wang, Z., Zhao, Z.X., Hong, N., Ni, D., Cai, L., Xu, W., and Xiao, Y. (2017). Characterization of causal agents of a novel disease inducing brown-black spots on tender tea leaves in China. *Plant Dis* 101(10), 1802-1811. doi:10.1094/PDIS-04-17-0495-RE

Xu, K.C., Zhang, R.Q., Li, J., Bai, Y.H., Yang, X.D., Sun, Y.X., and Huang, Q. (2019). *Camellia sinensis*, a new host plant of *Ceratocystis fimbriata* from China. *Plant Dis* 103, 2670. doi:10.1094/pdis-04-19-0802-pdn

Zhou, L.X., and Xu, W. (2014). First report of *Alternaria alternata* causing leaf spots of tea (*Camellia sinensis*) in China. *Plant Dis* 98(5), 697. doi:10.1094/PDIS-10-13-1086-PDN
